# Adherence to human lung microvascular endothelial cells (HMVEC-L) of *Plasmodium vivax* isolates from Colombia

**DOI:** 10.1186/1475-2875-12-347

**Published:** 2013-09-30

**Authors:** Briegel De las salas, Cesar Segura, Adriana Pabón, Stefanie CP Lopes, Fabio TM Costa, Silvia Blair

**Affiliations:** 1 Grupo Malaria, Facultad de Medicina, Universidad de Antioquia UdeA, Calle 70 No. 52-21, Medellín, Colombia; 2 Programa de Biología, Facultad de Ciencias Básicas, Universidad del Atlántico 080001, Barranquilla, Colombia; 3 Departamento de Genética, Evolução e Bioagentes, Universidade Estadual de Campinas (UNICAMP), Campinas, SP, Brazil

**Keywords:** Cytoadherence, *Plasmodium vivax*, Malaria, ICAM-1, Microvascular line of human lung endothelium, Colombia

## Abstract

**Background:**

For years *Plasmodium vivax* has been considered the cause of benign malaria. Nevertheless, it has been observed that this parasite can produce a severe disease comparable to *Plasmodium falciparum.* It has been suggested that some physiopathogenic processes might be shared by these two species, such as cytoadherence. Recently, it has been demonstrated that *P. vivax*-infected erythrocytes (*Pv*-iEs) have the capacity to adhere to endothelial cells, in which intercellular adhesion molecule-1 (ICAM-1) seems to be involved in this process.

**Methods:**

Adherence capacity of 21 Colombian isolates, from patients with *P. vivax* mono-infection to a microvascular line of human lung endothelium (HMVEC-L) was assessed in static conditions and binding was evaluated at basal levels or in tumor necrosis factor (TNF) stimulated cells. The adherence specificity for the ICAM-1 receptor was determined through inhibition with an anti-CD54 monoclonal antibody.

**Results:**

The majority of *P. vivax* isolates, 13 out of 21 (61.9%), adhered to the HMVEC-L cells, but *P. vivax* adherence was at least seven times lower when compared to the four *P. falciparum* isolates. Moreover, HMVEC-L stimulation with TNF led to an increase of 1.6-fold in *P. vivax* cytoadhesion, similar to *P. falciparum* isolates (1.8-fold) at comparable conditions. Also, blockage of ICAM-1 receptor with specific antibodies showed a significant 50% adherence reduction.

**Conclusions:**

*Plasmodium vivax* isolates found in Colombia are also capable of adhering specifically *in vitro* to lung endothelial cells, via ICAM-1 cell receptor, both at basal state and after cell stimulation with TNF. Collectively, these findings reinforce the concept of cytoadherence for *P. vivax*, but here, to a different endothelial cell line and using geographical distinct isolates, thus contributing to understanding *P. vivax* biology.

## Background

It has been estimated that *Plasmodium vivax* causes around 80–300 million cases per year, mainly in the Asian and Latin American regions [1]. For a long time *P. vivax* was considered the cause of benign malaria [1]. Nevertheless, it has been observed that this species can cause severe disease similar to *Plasmodium falciparum* and complications caused by *P. vivax* mono-infection have been described, such as cerebral malaria with generalized seizures and epileptic status, severe anaemia, hepatic dysfunction and jaundice, acute lung lesion, acute respiratory distress syndrome (ARDS) and pulmonary oedema, shock, splenic rupture, acute renal failure and acute thrombocytopaenia with or without bleeding on different body sites [2-9]. Most recently, goes back to 2008 with the acquisition of *P. vivax* genomic sequence, it was found that around 80% of the genes are orthologous and mostly synthenic between the *P. vivax* and *P. falciparum* genomes [10-12]. Taken together this suggests that *P. falciparum* malaria pathogenic processes related to cytoadhesion may occur in *P. vivax*[13]. Furthermore, studies performed by Anstey *et al.* suggested that pulmonary lesions found in *P. vivax* malaria could be caused by sequestration of parasitic forms within this organ [14]. In this sense, Carvalho *et al.* reported that *P. vivax* has the ability to cytoadhere *ex vivo* to human lung endothelial cells (HLEC) and, to a lesser extent, to placenta cryosections. The adhesion found was similar in strength to that observed in *P. falciparum*, though ten times lower in number [13]. Accordingly, Chotivanich *et al.* demonstrated that *P. vivax* isolates from Thailand have the ability to adhere to chondroitin sulphate A (CSA) and to hyaluronic acid (HA) in placental sections [15-18]. Moreover, in 2012, Bernabeu *et al.* reported the participation of *vir* 14 protein in the adherence to ICAM-1 receptor on the Chinese hamster ovary (CHO) cells transfected with human ICAM-1 (CHO-ICAM-1) cell line [18].

In Colombia, *P. vivax* continues to cause high impact on public health, with 83,255 (70.7%) cases in 2010, from a total of 117,650 reported malaria cases. Severe *P. vivax* malaria cases have been described primarily in newborn, children and pregnant women [9,19,20]. Due to the emergence of complications caused by *P. vivax* infection and its cytoadherence capacity, the purpose of this study was to determine the adherence capacity of *Pv-*iEs from Colombian patients, to human lung endothelial cells (HMVEC-L) mediated by ICAM-1 receptor in static conditions.

## Methods

### Ethical considerations

This study was approved by the Faculty of Medicine of the University of Antioquia’s ethics committee records 012 – 18 June, 2009. All participants agreed to voluntarily donate their blood samples after signing an informed consent.

### Study area and subjects

Up to 8.5 ml of peripheral blood was collected from 21 *P. vivax* non-complicated malaria patients, diagnosed by the thick-smear method and confirmed by rapid diagnostic test (Bioline SD05FK80) and polymerase chain reaction (PCR) (to dismiss mixed infections at low levels). Seven patients, all from different places of the Department of Antioquia, were recruited at the University of Antioquia Malaria Group office in Medellin, and the remaining 14, at the Nuestra Señora del Carmen Hospital in El Bagre – Bajo Cauca- Department of Antioquia (7° 35′ 0″ N, 74° 48′ 0″ W). To compare the *P. vivax* isolates adhesion, four *P. falciparum* isolates were adapted to *in vitro* culture [21].

### Isolation and concentration of *Plasmodium vivax* mature forms

**Figure 1:**
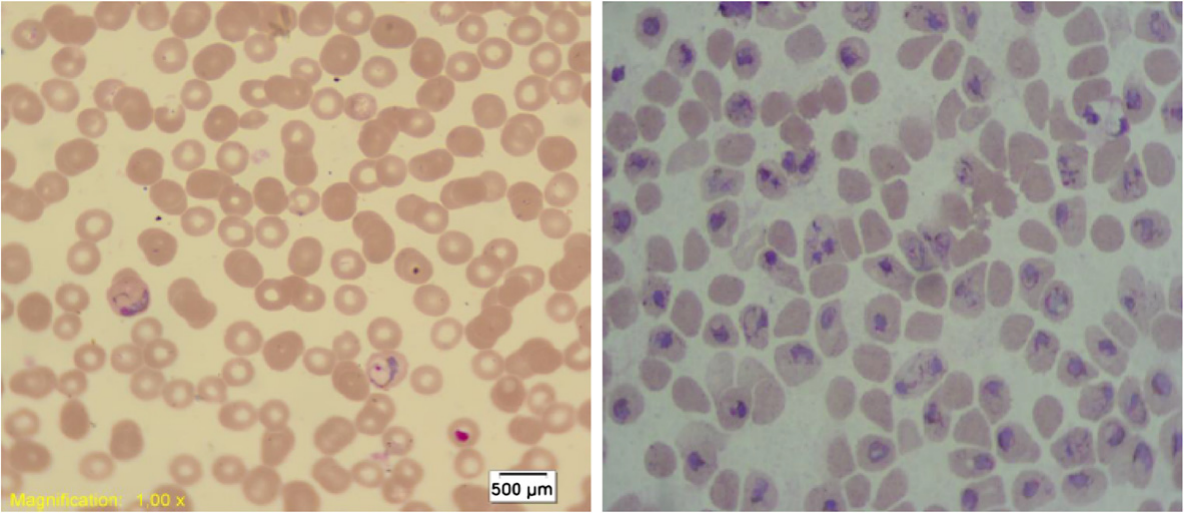
**Percoll concentration, before and after enrichment 45% Percoll gradient concentration of the mature stages of *P. vivax*.** 1% Giemsa stain of trophozoites, schizonts and gametocytes. 100× objective.

The methodology described by Carvalho *et al.*[13] was followed with few modifications. The initial patient parasitaemia was determined by counting the different parasitic forms in 200 leucocytes with an 8,000 leukocytes standard in order to obtain the amount of parasites per μL. Samples were processed immediately after blood collection [22]. Plasma was removed by centrifugation at 2,500 rpm; for removal of WBC, blood was filtered with a Whatman CF11 cellulose column, the resultant pellet was washed three times using RPMI 1640 (Sigma) pH 7.2 medium, and resuspended to a 10% haematocrit. Later, the *Pv-*iEs with mature forms (trophozoites, schizonts and gametocytes) were concentrated and separated from the young stages (rings) and non-infected erythrocytes. For this purpose, 5 mL of the 10% erythrocytes suspension were overlaid on 5 mL of 45% Percoll solution (Sigma P1644) and then the mixture was centrifuged at 2,500 rpm for 20 min at 4°C. The interphase containing mature forms was washed three times with RPMI-1640 pH 7.2. Mature stages were observed by thin smears (Figure 1). The estimated percentage of mature forms and the number of *Pv-*iEs per ml was obtained using the Neubauer chamber count (0.0025 sq mm).

### Cultivation of *Plasmodium falciparum* blood forms

*Plasmodium falciparum* FCR3/Gambia strain (donated by National Institutes of Health- NIH, Laboratory of Malaria and Vector Research) were cultured following Trager and Jensen’s method [21]. Mature stages concentration was carried out using 60% Percoll [23].

### Isolation and concentration of *Plasmodium falciparum* mature forms harvested from infected patients

The four field *P. falciparum* isolates were adapted to an *in vitro* culture and were maintained as described for FCR3 strain. The mature concentration stages were obtained with 1% Gelatin flotation (Sigma G2625) [24]. The percentage of mature forms, and the number of *P. falciparum*-infected erythrocytes by ml were estimated using the Neubauer chamber count, as previously described [25].

### Human lung endothelial cells (HMVEC-L) culture

The HMVEC-L was maintained following the distributor’s protocol (Lonza CC-2527), in endothelial basal medium -2 (EBM-2) (CC-3156) supplemented with endothelial growth medium (EGM-2) MV BulletKit from Lonza-Clonetics (CC-3202) at 37°C and 5% CO_2_, until achieving a 70-90% confluence. The cells were then treated with Trypsin-EDTA (Lonza) and seeded in Lab-Tek II 8 wells chambers (Brand-Products).

### *Plasmodium vivax* cytoadherence assay in static conditions

Cytoadherence assays in static conditions were performed according to Carvalho *et al.*[13]. Briefly, HMVEC-L were seeded in Lab-Tek II chambers (Brand-Products), and 5 × 10^4^ Percoll-enriched parasites per well were incubated in a total volume of 200 μL cytoadhesion medium (RPMI-1640, pH: 6.8) at 37°C for one hour in incubator agitation at 90 rpm (IKA KS 4000 IC Control). Non-adhered parasites and non-infected erythrocytes were removed by extensive washing in cytoadhesion medium. The adhered parasites were fixed using methanol for 2 min, and stained with Giemsa 1%. The number of parasites adhered were counted in a total of 200 HMVEC-L.

### ICAM-1 (CD54) expression in HMVEC-L treated with TNF

The ICAM-1 expression in HMVEC-L was induced by TNF treatment (10 ng/mL; Sigma-Aldrich) for 18–22 hours. The ICAM-1 expression levels evaluation was determined in a FACS Canto II flow cytometer (BD Biosciences) using a Mouse Anti-Human CD54 monoclonal (FITC-Conjugated, Millipore) for 30 min, at 4°C in approximately 500,000 cells. The fluorescence intensity and percentage of positive cells for ICAM-1 was analysed in a minimum of 10,000 events acquired and analysed using the WinMDI 2.8 program. The adherence specificity to the ICAM-1 receptor was performed by previous incubation of HMVEC-L TNF stimulated cells with 10 μg/ml of anti-human CD54 monoclonal (clone 15,2 Serotec AbD MCA532) at room temperature for 30 min and then cytoadherence assay were performed as described.

### Statistical analysis

The statistical significance of the performed comparisons was determined using the Mann–Whitney *U* test. Calculations were performed using the IBM-SPSS Statistics 21 program, and the charts with the Prism Software (6.01 version; GraphPad Software). Differences were considered significant when a p < 0.05.

## Results

**Figure 2:**
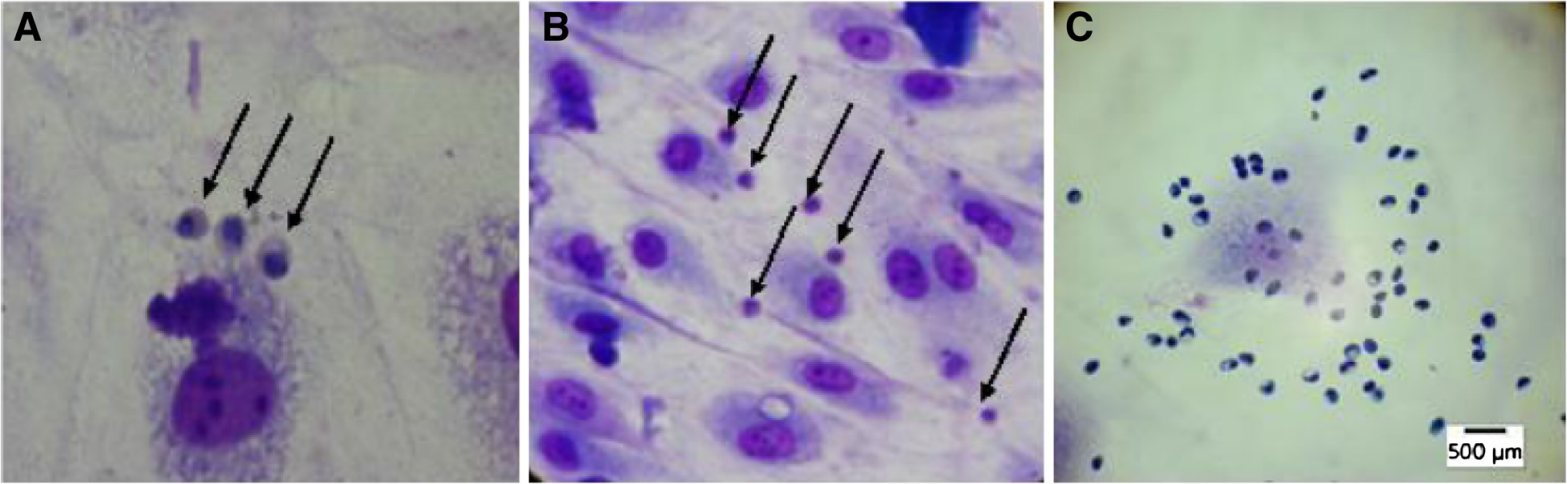
**Cytoadherence of clinical isolates of *Plasmodium sp. *to human lung endothelial cells HMVEC-L A and B.** Cytoadherence of *Pv*-iEs to human lung endothelial cells HMVEC-L. 1% Giemsa stain. **C**. Cytoadherence of *Pf*-iEs to human lung endothelial cells HMVEC-L. A 100×, B 40× and C 40× objective.

A total of 21 *P. vivax* isolates confirmed through PCR were included. Average parasitaemia was 7,604.7 ± 6,963 parasites/μL. These isolates came from different regions of Antioquia, mainly from Bajo-Cauca, and presented >60% mature asexual stages predominance. The average age of the 21 patients was 28.4 ± 14.1 years and 15 (71.4%) of them were males. The average parasitaemia of the adherent isolates was 9,461.5 ± 4,280.6 parasites/μL (Table 1). Thirteen *P. vivax* isolates (61.9%) presented adherence to HMVEC-L both at basal state and when cells were stimulated with TNF. The average basal state of *Pv*-iEs per 200 HMVEC-L was 32.5 ± 19.1; and after TNF stimulation a 1.6-fold increasing (52.1 ± 19.5, p *<* 0.001) in parasite-infected erythrocytes was observed (Table 2) (Figure 2A and B). No significant statistical differences (p > 0.05) between the parasitaemia averages from adherent and non-adherent parasites were noticed.

**Table 1:** **Percentage of cytoadherence of *Plasmodium vivax *isolates**

		**Frequency**	**Percentage**	**Average parasitaemia parasites/μL (SD)**	**Significance (95%)**
***P. vivax*isolates**	**Adherent**	13	61.9	9,461.5 (7766.1)	0.122
	**Non-adherent**	8	38.1	4,587.5 (4280.6)	
	**Total**	21	100.0		

Total cytoadherence of *P. vivax* isolates*,* average parasitaemia and their standard deviations, significance value 95%.

**Table 2:** **Cytoadherence of *Plasmodium vivax *and *Plasmodium falciparum *isolates in HMVEC-L basal and stimulated with TNF-α**

			**Adherence (% of inhibition)**
**Isolate**	**Adherence**^**1**^		**Anti-CD54**
***P. vivax***	**Basal**	**Stimulated TNF-α**	
***(*code*)***			
**0.012**	19.0 ± 1	27.0 ± 2	ND
**0.013**	19.5 ± 2.5	39.5 ± 5.5	ND
**0.014**	10.5 ± 1.5	33.5 ± 3.5	ND
**0.015**	4.0 ± 1	24.5 ± 2.5	ND
**0.017**	42.0 ± 4	51.0 ± 16	ND
**0.018**	28.0 ± 3	52.0 ± 7	31.5 (39.4)
**0.019**	38.0 ± 9	58.5 ± 6.5	34 (41.9)
**0.022**	37.0 ± 11	81.5 ± 2.5	36.5 (55.2)
**0.024**	53.0 ± 6	77.5 ± 10.5	33.5 (56.8)
**0.025**	28.5 ± 6.5	67.5 ± 3.5	23.5 (56.8)
**0.026**	55.5 ± 7.5	69.5 ± 11.5	27 (61.2)
**0.027**	35.5 ± 10.5	47.0 ± 8	42 (10.6)
**0.028**	22.0 ± 4	48.5 ± 4.5	22 (54.6)
***P. falciparum***			
**0.033**	58.5 ± 13.4	113 ± 16.9	
**0.034**	17 ± 7.1	53 ± 8.5	
**0.035**	475 ± 38.2	1050 ± 74.9	
**0.036**	97 ± 8.5	259 ± 24	
**FCR3**	201 ± 49.8	465 ± 61	

*P. vivax* and *P. falciparum* isolates cytoadherence to microvascular line of human lung endothelium HMVEC-L, from the isolate code 0.018 – 0.028 the percentage of adherence inhibition is described, compared to endothelial cells treated with TNF.

^1.^ Average ± standard deviation of *Pv-*iEs per 200 HMVEC-L.

Adherence of four *P. falciparum* isolates at basal state was 161.8 ± 196.2 and with TNF stimulation adhesion reach to 368.7 ± 429.1 (1.8-fold) and the adherence observed in the *P. falciparum* strain (FCR3) was 201 ± 61 parasite infected erythrocytes and after TNF stimulation it was 465 ± 498 (Table 2) (Figure 2C).

**Figure 3:**
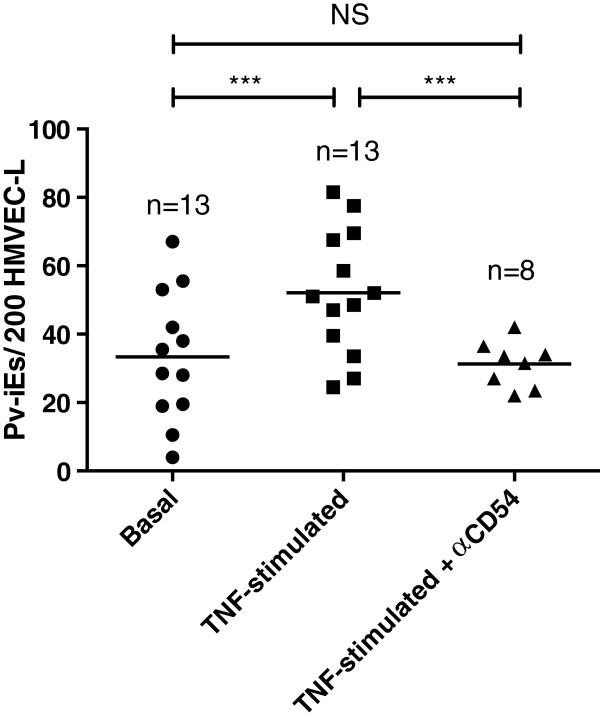
**Average adherence of*Pv*-iEs to the ICAM-1 endothelial receptor.** 5*10^4^ parasites/well were added to HMVEC-L-TNF, HMVEC-L-TNF- antiCD54 mAb, or to the basal control HMVEC-L. The scatter plot shows adherence data is an average of the number of *Pv*-iEs/200HMVEC-L; Asterisks indicate statistical significance with respect to adhesion values, as follows: *0.05 > p > 0.01; **0.01 > p > 0.001; ***p < 0.001.

The involvement of the ICAM-1 receptor (CD54) in parasite cytoadherence was determined in eight *P. vivax* isolates through cytoadherence inhibition assay using the monoclonal antibody anti-CD54 in TNF stimulated cells. A 50% cytoadherence reduction was observed in the average of adhered *Pv*-iEs/200 HMVEC-L, compared to cells without antibody anti-CD54, 62.7 ± 19.5 *vs.* 31.2 ± 8.8 *Pv*-iEs (Figure 3).

## Discussion

Cytoadherence of *P. vivax* isolates from Colombia was demonstrated using primary microvascular endothelial lung cells in static conditions as respiratory distress are one of the most frequently malaria vivax complication reported [26-29]. These assays revealed that the majority of *P. vivax* isolates, 13 out of 21 (61.9%), adhered to the lung endothelial cells and stimulation with TNF lead to a significant increase of 1.6-fold in *P. vivax* cytoadherence. Moreover, blockage of ICAM-1 receptor with specific antibodies showed a 50% significant reduction in parasite binding, thus demonstrating a *P. vivax* ability to adhere to the ICAM-1 receptor. As expected, *P. vivax* adherence was at least seven times lower in comparison to *P. falciparum* isolates. This latter finding is consistent with a previous report using Brazilian isolates [13].

The involvement of TNF in malaria pathogenesis has already been demonstrated in experimental cerebral malaria model [30]. The TNF released by monocytes in response to the endothelium damage or in the presence of soluble factors derived from the parasite such as glycosyl-phosphatidylinositol (GP-I) and haemozoin, increases ICAM-1 levels expressed in endothelial cells, and therefore enhances leukocytes and platelets adhesion and parasite sequestration to the endothelium [30,31]. Moreover, inflammatory co-morbidities, including bacterial infections, seem to play a key role in *P. vivax* clinical complications [3]. Therefore, endothelial cells were treated with TNF prior to *Pv*-iEs adhesion as demonstrated. These results suggest that in an ICAM-1 overexpression scenario due to high expression of TNF allowed *P. vivax* to improve its adhesion ability. In order to explore this hypothesis, the ICAM-1 receptor was blocked using anti-CD54 showing an average reduction of 50% in the cytoadherence in TNF stimulated cells; even in some of the isolates was below basal levels. This demonstrates its role in cytoadherence and suggests the participation of other endothelial receptors, such as hyaluronic acid (HA) and chondroitin sulphate A (CSA). *Plasmodium vivax* adherence to ICAM-1 receptor has already been suggest by Carvalho *et al.*, who demonstrated a significantly higher *Pv*-iEs adherence to CHO ICAM-1 transfected cells in comparison to mock CHO. The findings by Bernabeu *et al.* indicate the involvement of VIR-14 protein in ICAM-1 cytoadherence [18].

Collectively, this study reinforces the recent findings about *P. vivax* ability to cytoadhere to endothelial cells and highlights the importance of ICAM-1 endothelial receptors in this phenomenon.

## Competing interests

All authors declare that they have no competing interests.

## Authors’ contributions

BD carried out laboratory work, analysed the data and helped to draft the manuscript. AP participated in the data analyses and helped to draft the manuscript. SB and CS conceived and coordinated the study. BD, AP, SCPL and FTMC design of the study and performed the statistical analysis. All authors read and approved the final manuscript.
